# Association of modified Nordic diet with cardiovascular risk factors among type 2 diabetes patients: a cross-sectional study

**DOI:** 10.15171/jcvtr.2018.25

**Published:** 2018-09-27

**Authors:** Elnaz Daneshzad, Shaghayegh Emami, Manije Darooghegi Mofrad, Sahar Saraf-Bank, Pamela J. Surkan, Leila Azadbakht

**Affiliations:** ^1^Department of Community Nutrition, School of Nutritional Science and Dietetics, Tehran University of Medical Sciences, Tehran, Iran; ^2^Department of Community Nutrition, School of Nutrition and Food Science, Isfahan University of Medical Sciences, Isfahan, Iran; ^3^Department of Biology, Faculty of Science, York University, Toronto, Ontario, Canada; ^4^Department of International Health, Johns Hopkins Bloomberg School of Public Health, Baltimore, Maryland, USA; ^5^Diabetes Research Center, Endocrinology and Metabolism Clinical Sciences Institute, Tehran University of Medical Sciences, Tehran, Iran

**Keywords:** Nordic Diet, Dietary Pattern, Cardiovascular Risk Factors, Type 2 Diabetes, Diabetic Patient

## Abstract

***Introduction:*** Cardiovascular disease (CVD) is one of the most important causes of mortality. Healthy diets can decrease CVDs and other chronic diseases especially in patients with type 2 diabetes. In this study, we investigate association between adherence to the modified Nordic diet and cardiovascular risk factors among patients with type 2 diabetes.

***Methods:*** This cross-sectional study was conducted among 339 type 2 diabetic patients. Anthropometric indices, blood pressure, and biochemical tests were evaluated. A validated and reliable semi-quantitative food frequency questionnaire (FFQ) was used to assess dietary intake. Nordic diet scores were calculated based on median intakes of six food groups.

***Results:*** Body mass index (BMI) was higher among participants who were in the lowest tertile of adherence to the Nordic diet (*P*=0.006). There was a significant association between socioeconomic status (SES) and adherence to the Nordic diet (*P*<0.0001). Participants who were in the top category of adherence to the diet had significantly lower levels of aspartate aminotransferase (AST) (*P*<0.0001). There was a significant inverse association between adherence to the Nordic diet and low density lipoprotein (LDL) levels (odds ratio [OR]=0.29 95% CI: 0.09, 0.91, *P*=0.025), high systolic blood pressure (SBP) levels (OR=0.35 95% CI=0.17-0.74, *P*=0.015), and risk of obesity (OR=0.25 95% CI: 0.10, 0.63, *P*=0.03).

***Conclusion:*** Results suggest that adherence to the Nordic diet is associated with reductions in the prevalence of obesity, LDL levels and blood pressure among type 2 diabetic patients. However, additional studies are needed to confirm these findings.

## Introduction

Type 2 diabetes usually results from reduced insulin secretion or decreased sensitivity to insulin. Diabetes is a chronic metabolic disease that impacts various dimensions of health.^1,2^ According to the World Health Organization (WHO), globally 300 million people will suffer from diabetes by 2025.^1^ The prevalence of type 2 diabetes is high in Middle Eastern countries such as Iran.^3^ In Iran, recent statistics show the prevalence of diagnosed diabetes among people between ages 25 to 64 years old was 7.7%.^4^ However, this is likely to be an underestimate, as there are also undiagnosed cases.^4^

Type 2 diabetes is a risk factor for cardiovascular disease (CVD), which is an important cause of death among type 2 diabetic patients.^5^ Blood glucose imbalance is also strongly related to increased risk of CVDs.^5^ Several environmental factors, including dietary intake, can affect the incidence of type 2 diabetes.^6^ Nutrition plays a key role in the prevention of type 2 diabetes and CVD risk factors. Common nutritional protocols for prevention of type 2 diabetes emphasize consumption of fruits, vegetables, low-fat dairy products and foods with reduced saturated fats.^5^ Currently, it is recommended that a person’s whole diet (in the form of a dietary pattern) be evaluated, rather than just one food or one food group.^2,7^ Indeed, detection of a protective dietary pattern to decrease type 2 diabetes is needed. The Nordic diet is a dietary pattern referring to consumption of traditional foods from the Nordic countries (the Scandinavian region), including whole grains, fruits (such as apples, pears, and berries), low-fat dairy products, fatty fish such as salmon, cabbage and root vegetables.^7,8^ Several components of the Nordic dietary pattern including whole grains, fruits and vegetables, and fish have been associated with lower risk of CVDs.^7^ The Nordic diet is based on organic foods and healthy fat, therefore it is lower in refined carbohydrates and foods lacking in nutrients and is rich in monounsaturated fats that promote heart health.^7^ Some studies have demonstrated beneficial effects of the Nordic diet on factors associated with the risk of type 2 diabetes as well as cardiovascular complications, such as body weight and insulin sensitivity. Despite some constrasting results, weight loss, reduced cholesterol, lipid profile, blood pressure, inflammation, and overall mortality, have generally been reported as resulting from compliance to this diet.^2,7,9,10^ While dietary patterns such as the Mediterranean diet and the DASH diet (dietary approaches to stop hypertension) are appropriate for glycemic control, modification of cardiovascular risk factors and prevention and treatment of type 2 diabetes, the Nordic diet also appears to be effective.^5^ Cultural and regional differences as well as level of food accessibility can lead to different dietary patterns in various populations and/or countries. Therefore, some Iranians adhere to dietary patterns similar to that of the Nordic diet.^11^ Given its widespread adoption and some of the beneficial effects of the Nordic diet, calls have been made for evaluating adherence to this diet and health status. While several review articles have focused on the relationship between diabetes and individual food items,^12^ the nature of the association between berry consumption and type 2 diabetes and between milk/milk product consumption and CVD or inflammatory markers, have been inconclusive.^12^ Processed and red meat consumption has shown increased CVD endpoints, but more nuanced studies are needed to evaluate variation across population subgroups.^12^ A cohort study among diabetic patients showed that grains were protective against type 2 diabetes and CVD.^13^ Also, a randomized controlled study conducted in patients with metabolic syndrome suggested that the Nordic diet was protective against type 2 diabetes.^2,12^ To the best of our knowledge, no research has been published regarding the association between the Nordic diet and CVD risk factors among diabetic patients. Based on the probable beneficial effects of this diet on those risk factors, we investigated the association between a modified Nordic diet and cardiovascular risk factors among patients with type 2 diabetes in Isfahan, Iran.

## Materials and Methods

This descriptive-analytical cross-sectional study included 339 type 2 diabetic patients recruited between 2014 and 2016 from several therapeutic centers in Isfahan catoring to diabetic patients. As these clinics serve all parts of Isfahan, participants represented the full range of socio-demographic characteristics. Cluster random sampling methods were used to select participants. Throughout the five sectors of Isfahan, we randomly chose two centers (or clinics) from each area. Based on previously calculated mean and standard deviations for BMI in this population,^8^ our target number of participants was 143 ([(z_1 -α⁄2_)^2^ xs^2^] ⁄d^2^= [(1.96)^2^ × (3.2)^2^]/[0.02×26.3]^2^= 143). However, in order to replace patients who were excluded due to under- or over-reported food intakes, we continued sampling until enrolling 339 individuals. Inclusion criteria included interest in participation, answering all of the questionnaire completely, a diagnosis of type 2 diabetes and age ≥30 years. Patients with other diseases such as thyroid disorders, CVD, liver and kidney disease, cancers as well as patients who were pregnant and lactating were excluded from this study. Type 2 diabetic patients who were referred to these centers and completed informed consent were selected. Patient dietary intake was assessed using a validated semi-quantitative food frequency questionnaire (FFQ). Information on medical history, liver, kidney, CVD, cancer and other health conditions was collected. Biochemical tests included high density lipoprotein (HDL) cholesterol, low density lipoprotein (LDL) cholesterol, triglyceride (TG), total cholesterol (TC), HbA1c, fasting blood sugar (FBS), aspartate aminotransferase (AST), fibrinogen and high-sensitivity C-reactive protein (hs-CRP). Anthropometric data including height, weight and waist circumference were recorded. Blood pressure data, physical activity and basic information such as age, sex, marital status, smoking, socioeconomic status (SES) (income, number of children, parental education, parental occupation, car ownership, housing) were collected. Participants ranged in age from 30 to 70 years old. The criteria for diagnosis of type 2 diabetes was fasting blood glucose ≥126 mg/dL (6.3 mmol/L).^14^ We also checked for patients whose caloric intake was <800 or >4200 kcal/d. However, no patients were out of this range, therefore no one was excluded on this basis.

### Anthropometric assessments

All participants were assessed for anthropometric measurements including height, body weight, body mass index (BMI) and waist circumference. Height was measured in the standing position, with shoes removed. Body weight was measured using digital scales while subjects were minimally clothed and were not wearing shoes. Waist circumference was measured where the waist was narrowest over light clothing. BMI was calculated as body weight (kg)/height^2^ (m). Systolic and diastolic blood pressure were measured three times by a digital instrument after participants sat for 15 minutes. The mean of these measurements was recorded.^15^

### Dietary assessments

First, to assess dietary intake in individuals during the past year, we used a 168-item semi-quantitative FFQ. Food analysis was done using Nutritionist IV software modified to reflect the Iranian context (First Databank Division, The Hearst Corporation, San Bruno, CA, USA). Validity and reliability of the FFQ have been reported previously showing good results.^16^

Because we failed to use the original Nordic diet score due to low consumption of food groups associated with original diet in this population, we created six groups based on the same micronutrient content. This modified Nordic diet score was based on: (1) rye and wholegrain breads with a median of 90, (2) oatmeal (chickpea, lentil, bean, oat, frumenty, soybean, split pea, vicia faba and mung bean) with a median of 20, (3) cabbages and vegetables (cucumber, lettuce, celery, tomato, zucchini, raw and boiled spinach, bell pepper and leafy vegetables) with a median of 132, (4) apples, pears and high antioxidant fruits (apple, apple juice, peach, strawberry, nectarine, pear, persimmon, apricot, dry apricot, mulberries, dry mulberries, plum and dry plum) with a median of 97, (5) root vegetables (potato, raw and boiled carrot, garlic, onion, and turnip) with a median of 41 and (6) fish (fish conserved in salt and oil and other fish) with a median of 2. We calculated the median consumption of these food groups according to the FFQ. Consumption above and below median intake were given 1 and 0 points, respectively. The score of each group was summed and were classified: 0-1 point for low adherence, 2-3 points for medium adherence and 4-6 points for high adherence.^7^

### Biochemical analysis

Blood samples were collected after 12 hours of fasting overnight. Separate tubes of sodium citrate buffers for plasma and serum were centrifuged at 4°C and 500 × g for 10 minutes. Tests that could be performed the same day were conducted immediately; otherwise, serum and plasma samples were immediately frozen (−20°C). FBS was measured using enzymatic reagents (Pars Azmoon, Iran). Serum AST was measured using commercially available enzymatic reagents (Pars Azmoon) on a BT-3000 (Biotechinica) auto-analyzer. High-sensitivity CRP (hs-CRP) ELISA was performed on serum (IBL International). Inter- and intra-assay coefficient variations (CV) were <5% for all measurements. We used standard and control solutions for all measurements and standard curves were plotted for all of these standardized measurements. The plasma fibrinogen level was assayed using the Clauss method, which quantitatively determines the concentration of fibrinogen by adding thrombin and recording the rate of fibrinogen conversion to fibrin. The laboratory staff were unaware of participant treatment status.

Serum triglyceride concentration was measured using triglyceride kits (Pars Azmoon Tehran, Iran) with glycerol phosphate oxidase using enzymatic colorimetric tests. Serum HDL-C was measured after precipitation of the apolipoprotein B containing lipoproteins with phosphotungstic acid. Serum LDL-C was measured using commercially enzymatic reagents (Pars Azmoon, Tehran, Iran). The inter- and intra-assay CV of all methods was <10%.

### Statistical analysis

To check for the normal distribution of all the variables, we plotted histogram curves and ran the Kolmogorov-Smirnov test. A score indicating adherence to the Nordic diet was calculated (as mentioned above in the Methods). Chi-square and one-way ANOVA tests were used to compare qualitative and quantitative characteristics of patients across different tertiles of adherence to the Nordic diet. Analysis of covariance was used to evaluate the association between dietary intakes and biochemical tests and adherence to the Nordic diet. All dietary intakes including the macro- and micro-nutrients, foods and food groups were adjusted for energy intake. Biochemical tests were tested through three models including a crude model, an age and sex-adjusted model (Model 1), and a model further adjusted for BMI and energy intake (Model 2). Binary regression was performed to evaluated associations between adherence to the Nordic diet score and CVD risk factors. CVD risk factors were defined based on the Adult Treatment Panel III (ATPIII) guidelines.^17^ All statistical analyses were performed using SPSS version 17 and <0.05 was considered a significant *P* value.

## Results

Among the 339 participants, the mean and standard deviation (SD) for age was 60.37 ± 13.20 years. Mean BMI was higher in participants in the lowest tertile of adherence to the Nordic diet (*P* = 0.006). There was a significant association between SES and adherence to the Nordic diet that revealed that levels of adherence to the Nordic diet differed by SES (*P* < 0.0001). All patient demographic characteristics are presented in Table 1. Dietary intakes of participants by the three categories of adherence to the Nordic diet are shown in Table 2. Participants with the highest levels of adherence to the Nordic diet consumed lower amounts of dietary carbohydrates (*P* < 0.0001), and higher amounts of protein, zinc, folate, potassium, calcium, phosphorus, magnesium, β-carotene, dietary fiber, and vitamin B2 and B6 (*P* < 0.0001), fat and vitamin B1 (*P* = 0.004), vitamin B3 (*P* = 0.007), vitamin B12 (*P* = 0.017), monounsaturated fatty acid (MUFA) (*P* = 0.019), iron (*P* = 0.001), and vitamin C (*P* = 0.005).

**Table 1:** Demographic characteristics of diabetic patients in different tertiles of the adherence to the Nordic diet (N = 339)

**Variables**	**All**	**Nordic diet adherence**			***P*** **value** ^a^
		**Low**	**Medium**	**High**	
**Number**	339	42	162	135	-
Sex (n)					
Male	217	24	102	91	0.446
Female	122	18	60	44	
Age (y)	60.37 ± 13.20	57.14 ± 15.16	60.83 ± 12.84	60.82 ± 12.94	0.239
Weight (kg)	70.88 ± 12.54	71.61 ± 13.23	68.70 ± 12.59	73.28 ± 11.88	0.007
		71.78 ± 1.89	68.87 ± 0.93	72.95 ± 1.04	0.013^b^
BMI (kg/m^2^)	25.57 ± 4.13	26.93 ± 5.39	24.88 ± 3.88	25.96 ± 3.85	0.006
		26.79 ± 0.63	24.93 ± 0.31	25.95 ± 0.34	0.010^b^
Waist Circumference (n)^d^					
Men >102 cm	16	1	4	11	0.084
Men ≤ 102 cm	201	22	97	80	
Women >88 cm	62	12	24	26	0.054
Women ≤88 cm	60	6	36	18	
Physical activity					
Low	65.8%	27	117	79	0.108^c^
Moderate	31.9%	15	41	52	
High	2.4%	0	4	4	
SES (n)					
Poor	19.5%	18	36	12	<0.0001
Moderate	66.1%	24	105	95	
Rich	14.5%	0	21	28	

Abbreviations: BMI: body mass index; SES: Socio-economic status Data are presented as Mean ± SD or number of individuals. ^a^ P values presented resulted from analysis of one-way ANOVA and Chi-square test for quantitative and qualitative variables. ^b^ P values presented resulted from ANCOVA analysis and were adjusted for age, sex, and SES. ^c^ P values resulted from Fisher exact test. ^d^ Mean WC was lower in highest tertiles of Nordic diet adherence, however, this was non-significant. Data are not shown.

**Table 2:** Dietary intakes of participants in different tertiles of the adherence to the Nordic dietary pattern scores (n = 339)

**Variables**	**Nordic diet adherence**			***P*** **value** ^a^
	**Low (n = 42)**	**Medium (n = 162)**	**High (n = 135)**	
Energy (kcal/d)	1296 ± 224.57	1429 ± 114.70	1787 ± 125.26	0.052
Carbohydrate (g/d)	273.1 ± 6.15^b^	257.2 ± 3.14	244.6 ± 3.44	<0.0001
Protein (g/d)	40.19 ± 2.53	47.63 ± 1.29	54.78 ± 1.41	<0.0001
Fat (g/d)	36.13 ± 1.80	39.41 ± 0.92	42.60 ± 1.01	0.004
Dietary fiber (g/d)	7.92 ± 0.75	9.08 ± 0.38	12.17 ± 0.42	<0.0001
PUFA (g/d)	11.45 ± 0.72	11.83 ± 0.36	12.70 ± 0.40	0.173
MUFA (g/d)	11.06 ± 0.76	12.07 ± 0.38	13.29 ± 0.42	0.019
SFA (g/d)	9.90 ± 0.71	11.14 ± 0.36	11.71 ± 0.40	0.086
Iron (mg/d)	11.42 ± 0.92	13.32 ± 0.47	15.25 ± 0.51	0.001
Magnesium (mg/d)	120.29 ± 8.51	153.66 ± 4.34	181.40 ± 4.76	<0.0001
Zinc (mg/d)	4.00 ± 0.28	4.96 ± 0.14	5.65 ± 0.16	<0.0001
Folate (mcg/d)	137.01 ± 13.57	176.57 ± 6.93	213.38 ± 7.59	<0.0001
Potassium (mg/d)	1486 ± 113.05	1878 ± 57.73	2262 ± 63.25	<0.0001
Phosphorus (mg/d)	573.4 ± 46.56	672.1 ± 23.78	795.4 ± 26.05	<0.0001
Calcium (mg/d)	560 ± 49.63	681 ± 25.34	795.1 ± 27.76	<0.0001
Vitamin C (mg/d)	73.24 ± 9.47	73.31 ± 4.83	95.81 ± 5.30	0.005
Vitamin E (mg/d)	10.25 ± 1.02	12.21 ± 0.52	13.09 ± 0.57	0.054
Vitamin B1 (mg/d)	1.18 ± 0.07	1.19 ± 0.03	1.36 ± 0.03	0.004
Vitamin B2 (mg/d)	0.93 ± 0.06	1.10 ± 0.03	1.27 ± 0.03	<0.0001
Vitamin B3 (mg/d)	13.04 ± 0.81	13.78 ± 0.41	15.43 ± 0.45	0.007
Vitamin B6 (mg/d)	0.64 ± 0.05	0.81 ± 0.02	0.97 ± 0.02	<0.0001
Vitamin B_12_ (mcg/d)	1.44 ± 0.18	1.85 ± 0.09	2.04 ± 0.10	0.017
α-Tocopherol (mg/d)	4.01 ± 0.34	4.08 ± 0.17	4.52 ± 0.19	0.181
Β-Carotene (mg/d)	112.10 ± 58.08	306.13 ± 29.66	550.21 ± 32.49	<0.0001
Rye/ wholegrain breads (g/d)	77.49 ± 15.08	127.0 ± 7.70	137.50 ± 8.44	0.003
Oatmeal (g/d)	11.50 ± 3.51	21.72 ± 1.79	34.34 ± 1.96	<0.0001
Cabbages/vegetables (g/d)	65.97 ± 56.89	135.30 ± 29.05	250.60 ± 31.83	0.004
Apples, pears/high antioxidant fruits (g/d)	70.38 ± 10.56	86.87 ± 5.39	131.8 ± 5.91	<0.0001
Root vegetables (g/d)	13.38 ± 5.29	42.94 ± 2.70	67.18 ± 2.96	<0.0001
Fish (g/d)	0.33 ± 1.06	4.52 ± 0.54	8.35 ± 0.59	<0.0001
Sweets (g/d)	35.18 ± 7.27	41.98 ± 3.71	43.94 ± 4.07	0.578
Processed foods (g/d)	0.01 ± 0.20	0.23 ± 0.10	0.32 ± 0.11	0.411
Red meat (g/d)	9.76 ± 1.42	6.49 ± 0.72	6.88 ± 0.79	0.119
Dairy products (g/d)	259.57 ± 35.77	308.82 ± 18.26	355.44 ± 20.01	0.044
Egg (g/d)	5.51 ± 1.34	8.25 ± 0.68	8.92 ± 0.75	0.086

Abbreviations: PUFA, poly-unsaturated fatty acids; MUFA, mono-unsaturated fatty acids; SFA, saturated fatty acids. Energy intake is presented as mean and SD. ^a^ Calculated by analysis of covariance (ANCOVA). ^b^ All values from this row are adjusted for energy intake and presented as mean and SE.

Means and SDs of biochemical tests in the crude and two adjusted models are displayed in Table 3. In the crude model, participants in the lowest category of the Nordic score had the highest TG (163.33 ± 71.41 vs. 161.99 ± 84.67 mg/dL; *P *= 0.03). However, after adjusting for sex, age, BMI, and energy intake, the association disappeared (*P* = 0.20). Participants in the top category of adherence to the Nordic diet had significantly lower levels of AST (31.41 ± 9.02 vs. 36.25 ± 16.38 mg/dL; *P* < 0.0001). The mean values of other biochemical tests did not significantly differ between the three categories of adherence to the Nordic diet (*P* > 0.05).

**Table 3:** Biochemical tests among diabetic patients based on different tertiles of the adherence to the Nordic dietary pattern scores (N = 339)

**Variables**	**All**	**Nordic diet adherence**			***P*** ** value** ^a^
		**Low**	**Medium**	**High**	
FBS (mg/dL)					
Crude model	111.24 ± 33.45	118.71 ± 35.77	110.22 ± 28.71	110.16 ± 37.69	0.304
Model 1		119.77 ± 95.17	110.12 ± 48.23	109.93 ± 52.83	0.214
Model 2		118.11 ± 94.62	111.07 ± 48.05	109.30 ± 52.65	0.327
SBP (mm Hg)					
Crude model	127.71 ± 17.50	130.95 ± 12.79	127.28 ± 18.83	127.22 ± 17.11	0.441
Model 1		131.04 ± 49.89	127.32 ± 25.22	127.14 ± 27.61	0.423
Model 2		130.55 ± 50.07	127.79 ± 25.04	126.86 ± 27.79	0.499
DBP (mm Hg)					
Crude model	127.71 ± 11.95	86.66 ± 11.45	82.83 ± 9.90	84.50 ± 14.08	0.146
Model 1		86.21 ± 33.50	82.91 ± 16.93	84.55 ± 18.59	0.209
Model 2		86.09 ± 33.87	83.04 ± 17.30	84.45 ± 18.77	0.290
TG (mg/dL)					
Crude model	151.78 ± 76.91	163.33 ± 71.41	140.28 ± 69.99	161.99 ± 84.67	0.031
Model 1		161.76 ± 217.97	140.32 ± 110.46	162.42 ± 121.13	0.032
Model 2		155.18 ± 209.50	144.53 ± 106.59	159.79 ± 116.35	0.202
HDL (mg/dL)					
Crude model	42.32 ± 9.70	41.71 ± 7.87	42.16 ± 8.95	42.70 ± 11.03	0.812
Model 1		41.31 ± 26.69	42.10 ± 13.43	42.89 ± 14.72	0.587
Model 2		41.69 ± 26.51	41.88 ± 13.43	43.07 ± 14.72	0.499
LDL (mg/dL)					
Crude model	94.17 ± 30.98	102.81 ± 29.51	93.98 ± 32.13	91.71 ± 29.73	0.127
Model 1		99.98 ± 83.76	94.24 ± 42.34	92.29 ± 46.39	0.338
Model 2		99.38 ± 84.31	94.75 ± 42.89	91.98 ± 46.76	0.354
TC (mg/dL)					
Crude model	181.53 ± 46.49	179.79 ± 33.59	185.84 ± 45.79	176.91 ± 50.42	0.249
Model 1		177.02 ± 129.97	185.94 ± 65.90	177.64 ± 72.16	0.235
Model 2		176.24 ± 130.89	186.13 ± 66.46	178.04 ± 72.71	0.235
AST (mg/dL)					
Crude model	32.64 ± 5.93	36.14 ± 6.56	32.88 ± 5.89	31.26 ± 5.29	< 0.0001
Model 1		36.23 ± 16.20	32.86 ± 8.28	31.26 ± 9.02	< 0.0001
Model 2		36.25 ± 16.38	32.76 ± 8.28	31.41 ± 9.02	< 0.0001
hs-CRP					
Crude model	2.96 ± 0.50	2.91 ± 0.52	2.99 ± 0.48	2.94 ± 0.51	0.602
Model 1		2.91 ± 1.28	2.99 ± 0.55	2.94 ± 0.73	0.589
Model 2		2.91 ± 1.28	2.99 ± 0.73	2.94 ± 0.73	0.507
Fibrinogen					
Crude model	247.30 ± 18.82	246.43 ± 14.56	248.40 ± 20.44	246.27 ± 18.01	0.594
Model 1		246.39 ± 53.94	248.42 ± 27.24	246.26 ± 29.82	0.586
Model 2		246.12 ± 54.30	248.23 ± 27.61	246.41 ± 30.19	0.663

Abbreviations: FBS: fasting blood sugar; SBP & DBP: systolic and diastolic blood pressure; TG: triglyceride; HDL & LDL: high and low-density lipoprotein; TC: total cholesterol; AST: aspartate trance aminase; hs-CRP: high sensitive-C reactive protein. Data presented as mean ± SD. ^a^ *P* values presented resulted from ANCOVA analysis and in crude model, model 1 which adjusted for sex and age, and model 2 which adjusted for model 1 + energy intake, and BMI.

The results of binary regression analysis are presented in Table 4. There was a significant association between adherence to the Nordic diet and the risk of having LDL levels >130 mg/dL, systolic blood pressure (SBP) >130 mm Hg, and risk of obesity. Participants with the highest tertile of adherence to the Nordic diet compared to those within the lowest tertile of adherence had 68% lower risk of LDL >130 mg/dL. After adjusting for sex, age, BMI, and energy intake, this association was significant (*P* = 0.02). Participants who were in the highest tertile of adherence to the Nordic diet compared to the lowest tertile of adherence had 65% lower risk for being hypertensive (SBP >130 mm Hg) after adjusting for age and sex. However, after adjusting for sex, age, BMI, and energy intake, this association was not significant (*P* = 0.22). Participants with the highest level of adherence to the Nordic diet compared to those with the lowest tertile of adherence had a lower risk of obesity (BMI more than 30 kg/m^2^) in all three models (*P* < 0.05).

**Table 4:** Odd ratios and 95% confidence intervals for different CVD risk factors according to tertiles of adherence to the Nordic dietary pattern (n=339)

**Variables**	**Nordic diet adherence**			***P*** trend*
	**Low (n = 42)**	**Medium (n = 162)**	**High (n = 135)**	
LDL ≥ 130				
Crude	1	0.57 (0.24-1.36)	0.32 (0.12-0.85)	0.020
Model 1^b^	1	0.76 (0.29-1.97)	0.41 (0.14-1.17)	0.074
Model 2^c^	1	0.63 (0.23-1.73)	0.29 (0.09-0.91)	0.025
TC ≥ 200				
Crude	1	1.60 (0.73-3.49)	0.91 (0.40-2.07)	0.294
Model 1^b^	1	2.10 (0.92-4.76)	1.35 (0.56-3.23)	0.914
Model 2^c^	1	1.63 (0.68-3.87)	0.87 (0.34-2.23)	0.332
TG ≥ 150				
Crude	1	0.48 (0.24-0.96)	0.64 (0.32-1.28)	0.627
Model 1^b^	1	0.50 (0.25-1.01)	0.68 (0.33-1.40)	0.778
Model 2^c^	1	0.47 (0.23-0.98)	0.61 (0.28-1.30)	0.692
FBS ≥ 110				
Crude	1	0.85 (0.43-1.69)	0.73 (0.36-1.48)	0.356
Model 1^b^	1	0.79 (0.39-1.59)	0.71 (0.34-1.47)	0.379
Model 2^c^	1	0.78 (0.38-1.61)	0.70 (0.32-1.50)	0.464
SBP ≥ 130				
Crude	1	0.45 (0.22-0.92)	0.40 (0.19-0.82)	0.032
Model 1^b^	1	0.42 (0.20-0.87)	0.35 (0.17-0.74)	0.015
Model 2^c^	1	0.52 (0.25-1.10)	0.51 (0.23-1.12)	0.225
DBP ≥ 85				
Crude	1	0.43 (0.21-0.85)	0.63 (0.31-1.28)	0.770
Model 1^b^	1	0.45 (0.22-0.92)	0.65 (0.32-1.34)	0.785
Model 2^c^	1	0.45 (0.22-0.93)	0.70 (0.33-1.50)	0.889
WC ≥ 88 (Women)				
Crude	1	0.33 (0.11-1.01)	0.72 (0.22-2.28)	0.834
Model 1^b^	1	0.36 (0.11-1.10)	0.72 (0.22-2.33)	0.893
Model 2^c^	1	0.46 (0.13-1.61)	1.09 (0.25-4.78)	0.937
WC ≥ 102 (Men)				
Crude	1	0.93 (0.10-8.79)	3.16 (0.38-25.79)	0.053
Model 1^b^	1	1.12 (0.11-10.87)	3.32 (0.39-28.30)	0.076
Model 2^c^	1	1.23 (0.12-12.48)	3.48 (0.39-31.01)	0.086
Obesity				
Crude	1	0.09 (0.04-0.23)	0.21 (0.10-0.47)	0.005
Model 1^b^	1	0.10 (0.04-0.24)	0.24 (0.10-0.54)	0.012
Model 2^c^	1	0.10 (0.04-0.25)	0.25 (0.10-0.63)	0.030

^a^ *P* values were calculated using logistic regression. ^b^ Model 1: adjusted for age, sex and energy intake. ^c^ Model 2: model 1 + socioeconomic status and physical activity. Risk of high metabolic factors was defined by ATPIII guidelines: (1) abdominal obesity (WC > 88 in women and >102 in men); (2) high serum triglycerides levels (≥ 150 mg/dL); (3) elevated blood pressure (≥ 130/85 mm Hg); (4) high serum LDL-C levels (≥ 130 mg/dL); (5) abnormal serum glucose levels (FBS ≥ 110 mg/dL); (6) high serum total cholesterol levels (≥ 200 mg/dL) (6) obesity (BMI ≥ 30)

## Discussion

To the best of our knowledge this cross-sectional study is the first to examine associations between the Nordic diet and cardiovascular risk factors among diabetic patients in Iran. The results showed significant associations between low adherence to the Nordic diet and several cardiovascular risk factors including high levels of LDL-C, SBP as well as obesity among patients with type 2 diabetes. However, there were no significant relationships between other CVD risk factors and adherence to the Nordic diet, which was perhaps related to cross-sectional nature of the study. Based on other research in the field of nutritional epidemiology, cultural and regional differences as well as food accessibility levels can lead to different dietary patterns across settings. As evidence of this, even in Iran individuals adhere to variants of the diet common in Nordic countries.^11^

According to our results, mean WC decreased with tertile of adherence to the Nordic diet, though this association was not statistically significant. However, the risk of obesity was lower in the highest tertile compared to lowest tertile of adherence. This association was attenuated after adjusting for age, gender, and energy intake, but remained statistically significant (*P* < 0.05). Clearly, energy intake as well as sex impact weight gain and obesity. Women have more fat mass compared to men due to their physiological and hormonal characteristics. Also, women generally are more concerned about their body weight and shape.^18^ Darwiche et al reported that a 12-week modified Nordic diet led to significant reductions in WC.^19^ Moreover, there was a significant association between adherence to the Nordic diet and reduction of SBP and LDL-C in our study. The significant association between SBP and adherence to the Nordic diet disappeared after adjusting for BMI and energy intake. Previous studies have shown a strong relationship between blood pressure and anthropometric indices such as BMI.^20^ Increased mass and higher BMI lead to peripheral resistance, increased blood volume and cardiac output, all of which leads to hypertension.^20,21^ Darwiche et al. have shown results inconsistent with those of our study in that they observed a reduction in blood pressure in after a 12-week Nordic diet intervention.^19^ Consumption of the Nordic diet components including vegetables, rye, barley, oatmeal, apple, and fruits can be effective for reducing high blood pressure. In the present study, dietary fiber and several dietary antioxidants were consumed in higher quantities among participants with the highest levels of adherence to the Nordic diet, which can play an important role in decreasing risk of high SBP and LDL-C. It is well known that these foods increase both dietary fiber and antioxidants.

It has been shown that 2 to 10 g of dietary fiber can reduce LDL-C concentrations.^22^ This has been attributed to the fact that dietary fiber reduce LDL-C by binding to bile acids and decreasing fat absorption.^22^ Fiber can control weight gain and obesity by reducing caloric density of food intake and also slowing the rate of food ingestion. Monitoring carbohydrate consumption and especially intake of more whole grains are other means to control diabetes, due to the effects of macronutrient composition (e.g. especially carbohydrate intake on glycemic index and glycemic load in patients with type 2 diabetes).^23^ Whole grains and legumes that contain fiber are inversely associated with obesity, CVD risk factors, and diabetes. Indigestible carbohydrates can reduce metabolic risk factors and hyperglycemia.^24,25^ Also, increased fiber requires more chewing that slows the rate of gastric emptying through appetite signals triggered by the nervous system, which lead to more satiety.^26^ Because of lower carbohydrate consumption, protein intake is thought to be increased in the Nordic dietary pattern.^19^ In line with this, in our study, we found protein consumption was increased among participants in the highest tertiles of adherence to this diet. Macronutrients are other food components that can slow the rate of gastric emptying.^27^

We observed a significantly lower risk of high LDL-C among patients in the highest tertile of adherence to the Nordic diet. However, the amount of fat intake was higher in the highest tertile of the Nordic diet. This association was significant even after adjustment for age, gender, BMI, and energy intake. These results are consistent with the those of Darwiche et al.^19^ Increased intake of n-3 MUFA and PUFA (n-3 mono- and polyunsaturated fatty acids) in the Nordic diet is effective for decreasing LDL-C. A Nordic diet that includes high amounts of seafood and fish is expected to have high amounts of omega-3 and unsaturated fatty acids and has cardioprotective effects by reducing the lipid profile.^28^ A limitation of this study is its cross-sectional design which prohibits making causal inferences. Case-control and cohort studies may be needed to confirm these findings. Also use of FFQs can result in under- or over-reporting of food intake. Moreover, because of low consumption of food groups associated with the Nordic diet in the Iranian population, we failed to create the original Nordic diet score and therefore created a modified score. A large number of participants and multicenter recruitment are strengths of this study.

In conclusion, it appears that adherence to the Nordic diet has considerable and beneficial effects on anthropometric indices and biochemical tests, however additional well-designed case-control studies or randomized controlled trials are needed to confirm these findings.

## Competing interests

There were no conflict of interest to declare.

## Ethical approval

This study approved by the Isfahan University of Medical Sciences (Protocol # 192040).

## Funding

Isfahan University of Medical Sciences funded and supported the present study.
